# Β-Cyclodextrin-graft-poly(amidoamine) dendrons as the nitric oxide deliver system for the chronic rhinosinusitis therapy

**DOI:** 10.1080/10717544.2021.1876183

**Published:** 2021-01-29

**Authors:** Tao Liu, Guowei Li, Xidong Wu, Shaohua Chen, Siyi Zhang, Hong Han, Hongbin Zhang, Xiaoning Luo, Xiang Cai, Dong Ma

**Affiliations:** a Department of Otolaryngology-Head and Neck Surgery, Guangdong Provincial People’s Hospital, Guangdong Academy of Medical Sciences, Guangzhou, China; b Department of Biomedical Engineering, Jinan University, Guangzhou, China; c Department of Pharmacology, Jiangxi Testing Center of Medical Instruments, Nanchang, China; d Department of Light Chemical Engineering, Guangdong Polytechnic, Foshan, China; e MOE Key Laboratory of Tumor Molecular Biology, Jinan University, Guangzhou, China

**Keywords:** Chronic rhinosinusitis, nitric oxide, star polymer

## Abstract

Chronic rhinosinusitis (CRS) is a rather prevalent condition with a chronic inflammatory process, which is hard to cure. Herein, a new antibacterial drug, nitric oxide (NO), was used for the attempt on CRS therapy. To achieve this, a star copolymer (β-CD-PAMAM) consisting of the β-cyclodextrin (β-CD) core and seven PAMAM-G3 arms, which was designed as a low-cytotoxicity and high NO loading carrier, were synthesized and characterizied. The obtained β-CD-PAMAM/NONOate showed the effect in inhibiting and dispersing the biofilm of *S. aureus*, as well as the effective antibacterial performance, implying the promising application in CRS treatment. The *in vivo* assay confirmed that β-CD-PAMAM/NONOate displayed excellent therapy effect on CRS and significantly improved the symptoms of the experimental rats, which was no significant different in therapy effect with the clinical Rhinocort. Incorporated with its little toxicity *in vitro* and *in vivo*, the β-CD-PAMAM/NONOate was suggested a promising application in CRS therapy.

## 1. Introduction

Chronic rhinosinusitis (CRS) is a rather prevalent condition with recent estimates demonstrating that roughly 5–12% worldwide is affected (Yim & Orlandi, 2020). The most recent consensus statements both from US and European authorities posit that current evidence suggests CRS is a chronic inflammatory process mediated by a complex interplay of environmental and genetic factors that are not fully elucidated, so there is a lack of therapeutic options for the mechanisms of CRS. What is certain that the pathogenic microorganism infection is a main factor (DeConde & Soler, 2016). Intranasal glucocorticoid spray is the preferred drug for the treatment of CRS, but there are some side effects when the spray is used for a long time. A minority of patients show local adverse reactions, such as nasal dryness, nasal bleeding and even nasal septal perforation (Schleimer, 2017). With higher dose, systemic effects of glucocorticoids may occur, like the growth retardation in children and adolescents. Meanwhile, the long-term safety of glucocorticoid needs to be further evaluated using. Other therapy strategies including saline irrigations, decongestants, leukotriene inhibitors, interleukin antibody, is slight or the overall evidence for their use in CRS remains insufficient (Orlandi et al., 2016; Patel et al., 2020). Therefore, there is clinically a pressing need to develop the safe, anti-inflammatory and biocompatible biomaterials.

Nitric oxide (NO) has been reported as a potent antibacterial agent against a broad spectrum of bacteria through the reaction with free radical superoxide (O_2_*^–^), which resulted in antibacterial agents like peroxynitrite (–OONO) and dinitrogen trioxide (N_2_O_3_) (McDougald et al., 2011). More importantly, NO is considered as the key regulator of biofilm dispersal, and the endogenous NO could induce the dispersal of the mature biofilm (Barraud et al., 2006; Namivandi-Zangeneh et al., 2018). Recent developments demonstrated that a low concentration of NO can induce the bacteria in biofilm back to the stage of susceptibility to antibiotics or antimicrobial agents. Furthermore, under the specific conditions NO can effectively irrigate bacteria in infected animal models with excellent biological safety (Barraud et al., 2012; Barnes et al., 2013; Yepuri et al., 2013; Wang et al., 2020). Therefore, the antibacterial and anti-inflammatory therapy combined with NO could be a promising strategy. However, as a free radical in the biological medium, NO can readily react with biomolecules, leading to its inactivation in vivo, Therefore, NO-releasing nanocarriers are considered an important platform able to spatiotemporally control NO release in different applications.

Up to now, three main types of NO donors have been designed for delivering exogenous NO into biological entities, including *S*-nitrosothiol (RSNO), nitroglycerin, and *N*-diazeniumdiolate (NONOate) (Yang & Schoenfisch, 2018). Due to its facile preparation and ability to release NO spontaneously under physiological conditions, the NONOate is now widely used as an efficient donor for NO delivery. However, for most of the current NONOate, due to the low NO loading capability, it remains challenging to induce the biofilm dispersal in order to achieve antibacterial effect, which severely limited their potential applications in clinical (Carpenter & Schoenfisch, 2012). To overcome this barrier, the total NO loading amount of the designed donors need to be high enough to inhibit the dynamic process of biofilm formation and destruct the formed biofilm, which requires a delicate chemical design of NO donors (Hetrick & Schoenfisch, 2006; Duong et al., 2014; Lu et al., 2015). Benefited from the highly dense dendritic architecture consisting of abundant secondary amine groups, poly(amidoamine) dendron (PAMAM) is an ideal NO donor and the resultant PAMAM-NONOate showed the improved NO loading amount with the increase of PAMAM generations (Yang et al., 2018). However, the cytotoxicity of PAMAM increases with the growth of its generations (Zhou et al., 2016). It has been reported that the functions of high generation, such as gene-delivery, and the low toxicity could be achieved spontaneously by the strategy to conjugate a number of low generation dendrons to a core molecule to form a star-shaped copolymer (Ma et al., 2014; Zeng et al., 2015; Zhou et al., 2018; Liu et al., 2020).

Herein, a star copolymer (β-CD-PAMAM) consisting of the β-cyclodextrin (β-CD) core and seven PAMAM-G3 (generation of 3) arms were synthesized. PAMAM was reacted with NO to form the NONOate donor. Besides the chemical structure characterization of the obtained β-CD-PAMAM/NONOate, its antimicrobial effect and the possible mechanism have also been explored and discussed. The obtained β-CD-PAMAM/NONOate showed the excellent antimicrobial effect against to *S. aureus* at a low concentration, and *in vivo* assays indicated that β-CD-PAMAM/NONOate could remarkably inhibit bacterial infection and effectively improve the treatment to CRS, suggesting a promising application in CRS therapy.

## 2. Materials and methods

### 2.1. Materials

β-CD (Aladdin Industrial Corporation, Shanghai, China) was recrystallized twice from distilled water and dried under the reduced pressure at 100 °C for 24 h. Propargylamine (99%), ethylenediamine, methanol, *N,N*-dimethylformamide (DMF) and methyl acrylate were obtained from Aladdin Industrial Corporation and distilled under the reduced pressure before use. Sodium azide (NaN_3_, 99%) was purchased from Alfa Aesar. Sodium ascorbate (99%), copper sulfate pentahydrate, triphenyl phosphine (TPP) and iodine (*I*_2_) were obtained from Aladdin Industrial Corporation and used as received. The bacteria strains *Escherichia coli* (*E. coli*) ATCC 25922 and Staphylococcus aureus (*S. aureus*) ATCC 29213 were obtained from Southern Medical University (Guangzhou, China). Tryptic soy broth (TSB) and lysogeny broth (LB) were purchased from Qingdao Hope Bio-Technology Co., Ltd. The LB agar slants were obtained from Beijing Solar Bioscience and Technology Co., Ltd and stored at 4 °C. High-pressure reactor (SLM-25) was purchased from Shanghai Yan Zheng Experimental Instrument Co., Ltd. NO gas was purchased from Gndgas (Zhaoqing, China). The LIVE/DEAD^®^ Baclight™ bacterial viability kit (L7012) was purchased from Thermo Fisher Scientific (Waltham, MA, USA). Dulbecco’s modified eagle’s medium (DMEM), fetal bovine serum (FBS), citrate buffer, phosphate buffered saline (PBS) and Dulbecco's phosphate buffered saline (DPBS) were purchased from Life Technologies Corporation. Total Nitric Oxide Assay Kit Griess reagent and Cell Counting Kit-8 (CCK-8) were purchased from Beyotime Institute of Biotechnology (Shanghai, China).

### 2.2. Synthesis of the star copolymer (β-CD-PAMAM)

**Scheme 1.**
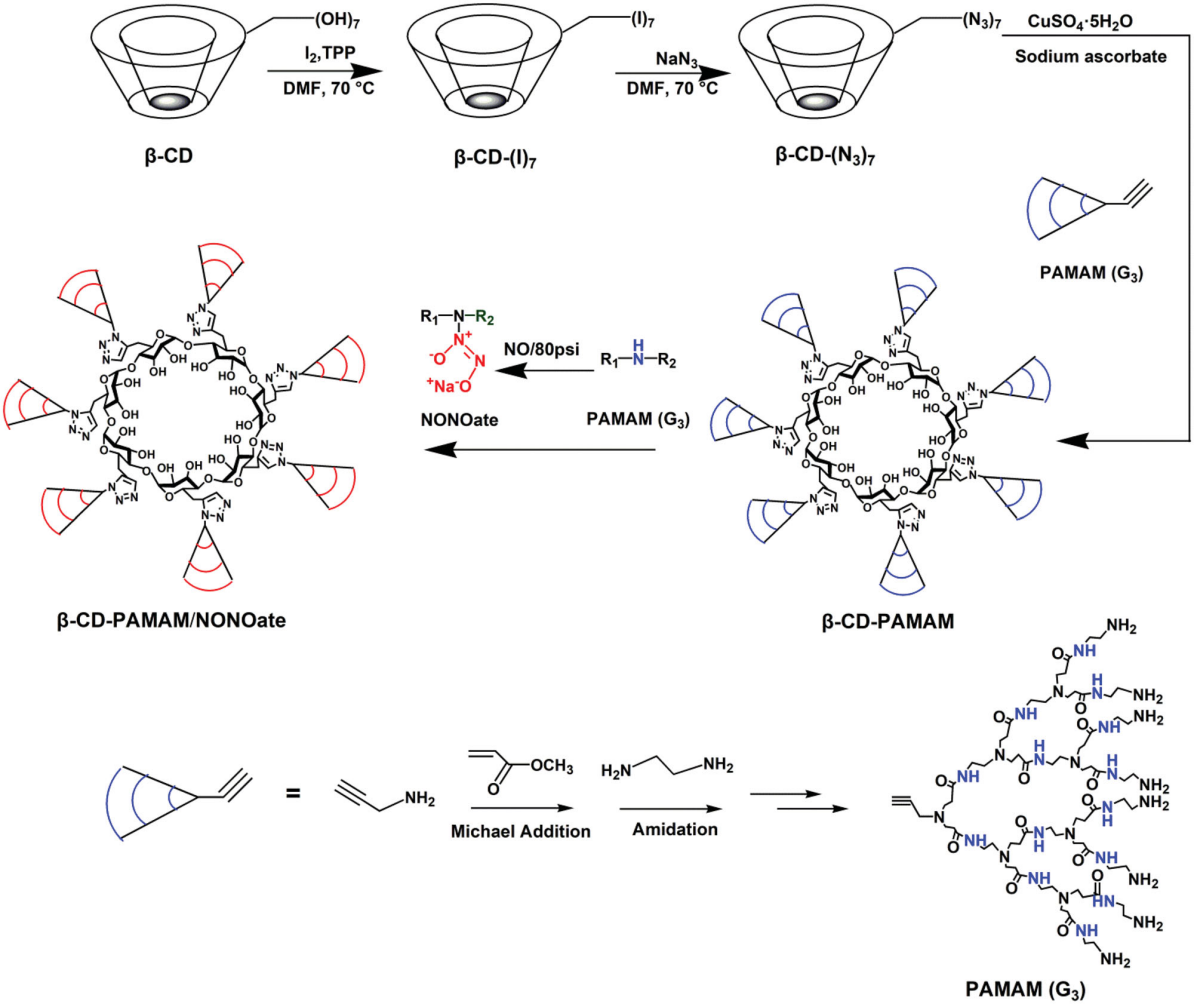
Synthesis routes of β-CD-PAMAM/NONOate.

The synthesis routes to β-CD-PAMAM were shown as Scheme 1, and the methods were performed according to the reported method (Wu et al., 2004; Deng et al., 2011; Liu et al., 2018). The detailed synthesis process and chemical structure characterization were shown in Supporting Information. The ^1^H NMR and FT-IR were carried out to confirm its chemical structure.

### 2.3. No loading and *in vitro* release

#### 2.3.1. No loading

The addition of *N*-diazeniumdiolate onto β-CD-PAMAM was carried out according to previous reports (Jin et al., 2018; Chen et al., 2019). β-CD-PAMAM/TCA was dissolved in dried tetrahydrofuran/methanol (v/v = 2:1) mixture and then placed in the high-pressure NO reactor with 20 psi nitrogen purging (10-15 s) for three times. Further nitrogen flushing (15 min) was followed by rapid fulfilling of 80 psi NO and the pressure was maintained for 3 d to complete the NO loading on β-CD-PAMAM. After the 3-day reaction of diazeniumdiolates functionalization, the reactor was flushed with 25 psi nitrogen for 30 min. The product was precipitated by adding an excess amount of cold ether, and the co-loaded system of β-CD-PAMAM/NONOate was obtained after vacuum drying and stored at −20 °C with sealing. The UV–Vis measurement was carried out by a UV-2450/2250 (Shimadzu) spectrophotometer with 3 independent scans. All tests were performed at 25 °C, and spectra over the range of 200–800 nm were recorded.

#### 2.3.2. No content in β-CD-PAMAM/NONOate

The NO content in β-CD-PAMAM/NONOate was determined through the Griess assay (Wo et al., 2016). Briefly, a certain amount of β-CD-PAMAM/NONOate was dispersed in citrate buffer (pH = 4.0) at 37 °C. Then, 50 μL β-CD-PAMAM/NONOate was mixed with 50 μL DPBS and 100 μL Griess reagent for 24 h at room temperature in the dark. After the centrifugation at 1000 rpm for 5 min, the absorbance of the upper layer was measured at 548 nm using the microplate reader. The NO concentration was calculated with the standard curve that we have established in our previous work (Yu et al., 2018) (*y* = 0.0052*x* − 0.0118, *R*^2^=0.999; where y is the OD_540_ value and *x* is the NO_2_^–^ concentration).

#### 2.3.3. *In vitro* NO release

For NO release assay, β-CD-PAMAM/NONOate (5 mg) was dispersed into PBS (5 mL) and then the mixture was enclosed in the dialysis bag (MWCO = 1000). The dialysis bag was immersed in 20 mL of PBS with mild stirring at 37 °C. At predetermined intervals, 5 mL of the release media was taken out and an equal volume of fresh PBS was added back. The released NO was evaluated using the above Griess regent determination, and all of the results were studied in triplicate.

### 2.4. Antimicrobial assays

#### 2.4.1. Bacteria preparation

Before antimicrobial assays, all samples and glassware were sterilized at 121 °C for 30 min. The bacteria were inoculated on TSB for Gram-positive *S. aureus* and LB for Gram-negative *E. coli* overnight at 37 °C with 150 rpm rotation. Then, 50 μL of the bacteria growth suspension was transferred into fresh 5 mL broth and incubated for another 4 h at 37 °C until the optical density (OD) value at 600 nm was about 0.1, a condition that the bacterial concentration was nearly 10^8^ colony (CFU/mL) (Lu et al., 2015). The bacterial suspensions were then centrifuged at 3500 rpm for 5 min and washed twice with PBS to remove the broth. Finally, the bacteria were re-suspended in a 0.9% stroke-physiological saline solution and adjusted to the required concentration.

#### 2.4.2. Biofilm inhibition assays

The laboratory strain Gram-positive *S.aureus* and Gram-negative *E.coli* were used to evaluate the effects of NO and TCA co-delivery system on the biofilm formation. The bacteria suspension in a 0.9% stroke-physiological saline solution was added into TSB or LB medium until the OD value at 600 nm was 0.005. Then, the bacterial medium was inoculated with β-CD-PAMAM/NONOate with the final concentration of 25 and 50 μg/mL. The samples treated with β-CD-PAMAM at 25 μg/mL as well as the untreated sample were set as control groups. The plates were incubated at 37 °C without shaking for 18 h. After the incubation, the supernatant was removed and the remaining biofilm was washed with PBS and then stained by 0.1% crystal violet dye. The plates were incubated on the bench for another 20 min before washing the wells twice with PBS. The amount of remaining crystal violet stained biofilm was quantified by adding 500 μL of ethanol and measuring OD_550_ value of the homogenized suspension.

#### 2.4.3. Biofilm dispersal assays

The bacteria in 0.9% stroke-physiological saline solution was firstly diluted to the desired level (OD_600_ = 0.1), then 100 μL of bacteria suspension was mixed with 900 μL broth (TSB for *S. aureus* and LB for *E. coli*) in a 24-well microtiter plate. Bacteria were incubated at 37 °C without shaking for 24 h. The culture medium was refreshed by every 12 h and the biofilm was allowed to form without any disruption. After that, β-CD-PAMAM/NONOate with the final concentration of 5-100 μg/mL was added to incubate for another 12 h. The samples treated with β-CD-PAMAM at 25 μg/mL as well as the untreated sample were set as control groups. After 12 h incubation, the culture medium was removed and the remaining biofilm at the bottom was washed with PBS. Then, each well was quickly fixed with 500 μL of methanol for 15 min at 4 °C, and 300 μL of 0.1% crystal violet dye was added to each well and the biofilm was stained for 30 min. Afterwards, the wells were washed twice with PBS again and dried at room temperature. The amount of remaining biofilm was quantified by adding 500 μL of ethanol and measuring OD_550_ value of the homogenized suspension. Photographs of the biofilms were captured using a digital camera.

#### 2.4.4. Fluorescence microscope and scanning electron microscopy

For fluorescence microscope observation, *S. aureus* and *E. coli* were grown in glass-bottom 24-well plates (NEST Biotechnology Co. LTD, China) with or without β-CD-PAMAM/NONOate at the beginning of growth as described above. After 12 h incubation, biofilms were washed with PBS and stained with SYTO-9 green fluorescent nucleic acid stain. The stained bacterial suspension (10 μL) was dropped to the glass slide and trapped by a cover slide. The confocal image was acquired by fluorescence microscope observation (Nikon-2000U, Japan). The green signal (SYTO-9) was observed by 488 nm Ar excitation laser.

For SEM observation, after 12 h incubation, the biofilm as described above was placed in 2.5 wt% glutaraldehyde solution for 12 h and then dehydrated in aqueous ethanol solution with the increasing ethanol concentrations until the specimen was placed in 100% ethanol. Afterwards, the specimen was dried and mounted on the scanning electron microscope stubs, coated with gold palladium and then examined with a Philips XL-30 scanning electron microscope (USA) at an accelerating voltage of 5 kV.

#### 2.4.5. Antibacterial activity

The bacteria in a 0.9% stroke–physiological saline solution was diluted to the desired level (1.5 × 10^7^ colony forming units, CFU/mL). Then, various formulations with different concentrations were added to co-incubate at 37 °C with 150 rpm rotation for 4 h. After that, bacteria suspensions were diluted by 100 times with PBS, and 100 μL of the diluent was spread onto LB agar plates and incubated at 37 °C for 12 h. The bacterial activities treated with different formulations were determined by counting the numbers of the colonies and capturing.

#### 2.4.6. Confocal microscopy observation

The bacterial suspensions were stained with live/dead baclight bacterial viability kits (L7012) according to the manufacturer’s protocol. *E. coli* and *S. aureus* were incubated in the same procedure mentioned above. After that, the bacterial suspension was centrifuged at 4000 rpm for 5 min and the resultant bacteria pellet was dispersed in 5 mL distilled water. Then, 1 mL of the bacteria pellet was transferred to 1.5 mL e-tube. The β-CD-PAMAM/NONOate and other control groups were added with a final concentration of 25 μg/mL. The bacterial was incubated at 37 °C for 4 h and then stained by L7012. The stained bacterial suspensions (10 μL) were then dropped to the glass slide and trapped by the cover slide. The confocal image was acquired by Leica SP8 Confocal Microscope (USA) and analyzed with Leica Microsystems. SYTO-9 green fluorescent nucleic acid stain was obtained by 488 nm Ar excitation laser, and the red signal propidium iodide (PI) was obtained by 543 nm He-Ne excitation laser.

### 2.5. *In vivo* assays

To establish the CRS model, fifty male SD rats weighing 300-400 g were selected, and the absorbable gelatin sponge with *S. aureus* suspension (0.3 mL, 1 × 10^9^ cells/mL) was introduced into their nasal cavities. The sponge was taken out after 3 days, and all procedures were performed twice a week with lasting 3 consecutive weeks to induce inflammation. Rhinosinusitis was defined by symptoms with purulent mucus and inflammatory cells were found in nasal mucous by the HE staining. The rats treated with the blank absorbable gelatin sponge were set as control. During the establishment of CRS models, rats with complications of lower respiratory tract infection were selected as experimental groups. After inoculation with *S. aureus*, some rats got symptoms of anorexia, reduced activities, sneezing and nasal rubbing, but no rats died.

After that, all animals were divided into five groups at random: the control, CRS group, CD-PAMAM group, β-CD-PAMAM/NONOate group and Rhinocort group. Rats in each group were given by intranasal administration in the morning and afternoon, respectively, every day. A treatment with three spray (approximately 0.09 mL each spray) in each nasal cavity, 2 s between each spray, for 14 consecutive days were performed for each rat. Rats of CRS group and the control group were given saline nasal administration. During the total period of drug treatment, their weight, diet, symptoms with nasal rubbing and nasal discharge were observed. After the drug treatment, rats were intraperitoneal injected of 5 mL/kg chloral hydrate (10%) and then the cervical dislocation was performed. The mandible was cut and fixed with 4% paraformaldehyde for 48 h. Under a dissecting microscope, the nasal cavity and sinus mucosa were stripped. Samples were subjected to dehydration, embedding, staining and paraffin sectioning. The changes of the body weight, diet, drinking water, nasal rubbing times and nasal discharge were all recorded.

### 2.6. Biocompatibility

#### 2.6.1. Cytotoxicity

For cytotoxicity study, primary mouse embryo fibroblasts (NIH 3T3 cells) were obtained from Guangzhou Southern Medical University and cultured in DMEM supplemented with 10% fetal bovine serum and 1% penicillin/streptomycin at 37 °C under 5% CO_2_ atmosphere.

The cytotoxicity of β-CD-PAMAM/NONOate was evaluated using the CCK-8 kit. NIH 3T3 cells were cultured onto a 96-well plate (8 × 10^3^ cells/well) with 100 μL complete culture medium in a humidified atmosphere incubator of 5% CO_2_ at 37 °C. After 12 h, β-CD-PAMAM/NONOate at different concentrations (0, 5, 10, 25, 50 and 100 μg/mL) was added into the 96-well plate and cultured with cells for another 24 h. After that, the cells were washed with PBS to eliminate the interference of β-CD-PAMAM/NONOate. All cells were exposed to the 10% (v/v) CCK-8 reagent in DMEM medium for 1 h at 37 °C, and the absorbance at 450 nm was recorded by a microplate reader. The cells treated with PBS and β-CD-PAMAM were set as control groups.

#### 2.6.2. *In vivo* toxicity

The major organs of the male SD rats including the heart, liver, spleen, lung and kidney were harvested from Section 2.5 and stained with H&E after the treatment, and all experimental operation were carried out as described above.

### 2.7. Statistical analysis

All data were expressed as the mean ± the standard deviation. GraphPad Prism 5 (GraphPad Software, Inc., La Jolla, CA, USA) was used to perform statistical analysis (one-way analyses of variance, ANOVA). The significance level was 0.05, and the data were indicated with * for *p* < .05, ** for *p* < .01 and *** for *p* < .001.

## 3. Results and discussion

### 3.1. Preparation and characterization of β-CD-PAMAM/NONOate

To deliver NO for CRS therapy, a star copolymer consisting of a β-CD core and seven PAMAM dendron arms was synthesized through a click reaction, and then NO was loaded by conjugating with the PAMAM arms. The synthetic routes to β-CD-PAMAM/NONOate were shown in Scheme 1.

**Figure 1.**
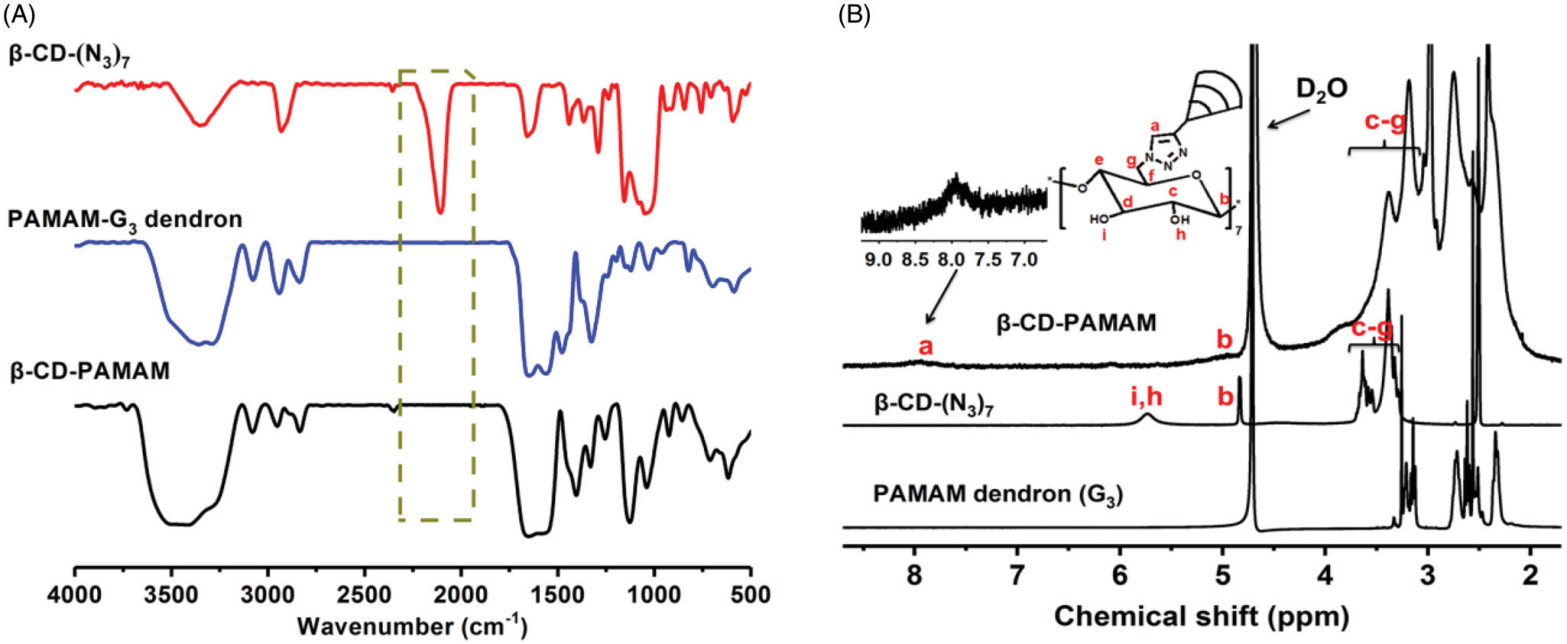
(A) FT-IR spectra of propargyl focal point PAMAM-G_3_, β-CD-(N_3_)_7_ and β-CD-PAMAM. (B) ^1^H NMR spectra of propargyl focal point PAMAM dendron (G_3_), β-CD-(N_3_)_7_ and β-CD-PAMAM.

Figure 1(A,B) confirmed the chemical structure of β-CD-PAMAM. As shown in Figure 1(A) of the FT-IR spectra, the propargyl focal point PAMAM-G_3_ dendron showed its characteristic absorption bands at 3280 cm^−1^ (ν_N–H_), 2937–2864 cm^−1^ (ν_C–H_), 1642 cm^−1^ (ν_C=O_) and 1557 cm^−1^ (ν_C=O–NH_). And per-6-azido-β-CD (β-CD-(N_3_)_7_) also showed its characteristic band at 2100 cm^−1^ (ν_N=N–N_). After the click reaction, the obtained β-CD-PAMAM showed the characteristic absorption bands of both PAMAM and β-CD-(N_3_)_7_. In particular, the band of azido at 2100 cm^−1^ disappeared, indicating that all azido groups of β-CD-(N_3_)_7_ were reacted completely with PAMAM and each β-CD conjugated with seven PAMAM arms. Figure 1(B) of ^1^H NMR spectra confirmed further the chemical structure of β-CD-PAMAM. As shown, for β-CD-PAMAM, the signal at 7.9 ppm indicated the presence of the triazole proton, which was attributed to the formation of β-CD-PAMAM by the Cu(I)-catalyzed Huisgen 1,3-dipolar cycloaddition of β-CD-(N_3_)_7_ with PAMAM (Ashton et al., 1996). Moreover, the integral ratio (*I*_a_:*I*_b)_ of the proton resonance signals from the triazole and the β-CD units was calculated as about 1:1, confirming further that each β-CD core conjugated with seven PAMAM arms to form a seven-arms star copolymer of β-CD-PAMAM.

**Figure 2.**
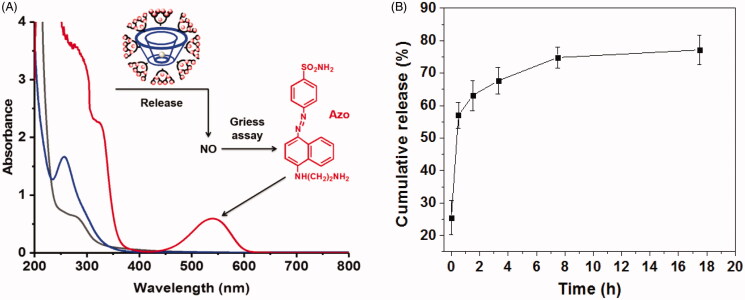
(A) UV − Vis absorption of β-CD-PAMAM (black line), β-CD-PAMAM/NONOate (blue line), and β-CD-PAMAM/NONOate treated with Griess assay (red line) at the concentration of 25 μg/mL in sodium citrate buffer (pH = 4.0). (B) The cumulative NO release profiles from β-CD-PAMAM/NONOate in PBS (pH = 7.4) at 37 °C (*n* = 3).

*N*-diazeniumdiolate (NONOate) was designed to deliver exogenous NO due to the facile synthetic procedure involving simple treatment of secondary amines of PAMAM with NO gas at 80 psi (Nguyen et al., 2016). Then, NO was reacted with the PAMAM segments and the delivery system of β-CD-PAMAM/NONOate was obtained. To confirm this and calculate the NO loading amount in β-CD-PAMAM/NONOate, UV–Vis was performed as Figure 2(A). Compared to blank β-CD-PAMAM, β-CD-PAMAM/NONOate in PBS showed an obvious absorption peak at about 250 nm, at which NONOate showed its characterized peak (Li et al., 2018). This result confirmed the successful loading of NO onto β-CD-PAMAM. As a NONOate donor, β-CD-PAMAM/NONOate could quickly release NO in an acid solution and then the released NO convert to NO^2–^ by the oxidation. The Griess assay could react with NO^2–^ and then appear purplish red color, so the Griess assay has been used widely as the NO indicator. It was found that the aqueous β-CD-PAMAM/NONOate treated with Griess assay showed the purplish red color and an appearance of a peak at 548 nm, indicating the released NO has been oxidized and then reacted with Griess assay. Moreover, the peak at 250 nm belonging to NONOate almost disappeared, indicating that almost all NO was released quickly in an acid solution. This result further confirmed that NO was loaded onto β-CD-PAMAM to form the β-CD-PAMAM/NONOate. The NO content in β-CD-PAMAM/NONOate was also determined through the Griess assay under an acid buffer for sufficient NO release. After calculating according to the standard curve, the NO content in β-CD-PAMAM/NONOate was determined as 11.65 μmol/mg, which was much higher than some other reports (Tan et al., 2012; Nurhasni et al., 2015). This result indicated that the star structure of β-CD-PAMAM was conducive to improve the NO loading amount for NONOate donors. Theoretically, the NO loading amount in β-CD-PAMAM/NONOate was 12.41 μmol/mg. The difference between the theoretical and actual NO loading may be resulted by the reaction efficiency and the steric hindrance of PAMAM segments.

For *in vitro* NO release assay, 1 mg β-CD-PAMAM/NONOate was trapped in a dialysis membrane with the MWCO of 1000 Da. The released NO was assessed also by Griess assay. As shown in Figure 2(B), NO could be released fast in an aqueous solution. Compared with that the half-life of only 3 s of NO released from small molecules (Hrabie & Keefer, 2002), the half life of NO released from β-CD-PAMAM/NONOate was about 30 min, and about 60% of NO released from β-CD-PAMAM/NONOate after an hour followed by further constant release lasting around 18 h. It has been reported that the density of primary amines at the exterior and the conformational freedom of the dendritic architecture of NO donors could enhance the diazeniumdiolate stability over small molecule primary amine substrates, resulting in the extended release period. PAMAM loaded NO to form the structure of *N*-diazeniumdiolate (NONOate), in which there is an initial burst of NO followed by slow and stable lasting release. As a matter of fact, the initial burst release of NO from β-CD-PAMAM/NONOate could result in the rapid dispersion of bacterial biofilms which could help the antibiotics enter and kill bacteria. After 30 h, the cumulative released amount of NO was about 11.5 μmol/mg, very nearly to the actual NO loading amount, which was considered that the loaded NO was fully released.

### 3.2. Antibiofilm activity

It is well recognized that most pathogenic bacteria can form the biofilm on a variety of surfaces, which is composed of microbial cells and their extracellular matrix accumulated on the surfaces. The biofilm is a physical barrier to resist the penetration of antibiotic agents, so the anti-biofilm activity of β-CD-PAMAM/NONOate was explored.

**Figure 3.**
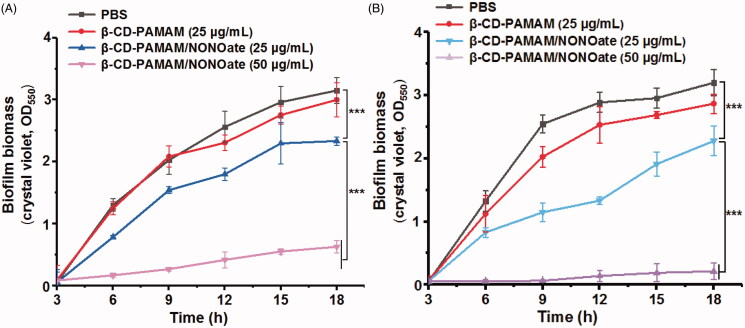
Inhibition results of biofilm formation for the bacterial of *E. coli* (A) and *S. aureus* (B). The data are presented as the mean ± standard deviations (***p < .001, *n* = 3).

The biological model of biofilm formation was then established to study the inhibition to biofilm formation of *S. aureus* and *E. coli*. As shown in Figure 3, β-CD-PAMAM showed some inhibition effect on biofilm formation due to its substantial amino groups, and there was a slight difference in biofilm biomass between β-CD-PAMAM and PBS control. After NO was loaded, β-CD-PAMAM/NONOate showed the obvious biofilm inhibition effect and the biofilm biomass decreased more than 20% compared with PBS control after 18 h. In particular, the inhibition effect was enhanced significantly by using a high concentration of 50 μg/mL, and 80% of biofilm for *E. coli* and 94% biofilm for *S. aureus* were inhibited compared with PBS after 18 h incubation, showing the outstanding biofilm inhibition effect of NO.

**Figure 4.**
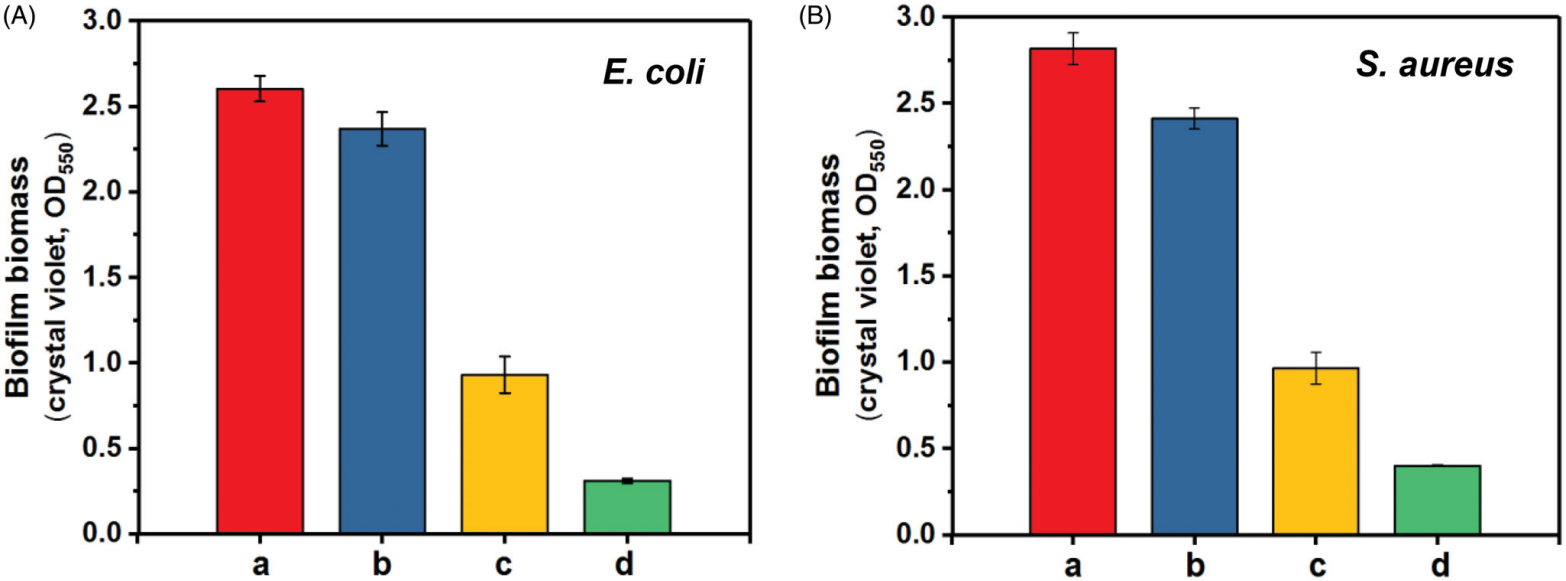
Biofilms of *E. coli* and *S. aureus* dispersion results treated with different formulations by crystal violet staining method (*n* = 3). (a: PBS control, b: 25 μg/mL blank β-CD-PAMAM, c: 25 μg/mL β-CD-PAMAM/NONOate, d: 50 μg/mL β-CD-PAMAM/NONOate).

**Figure 5.**
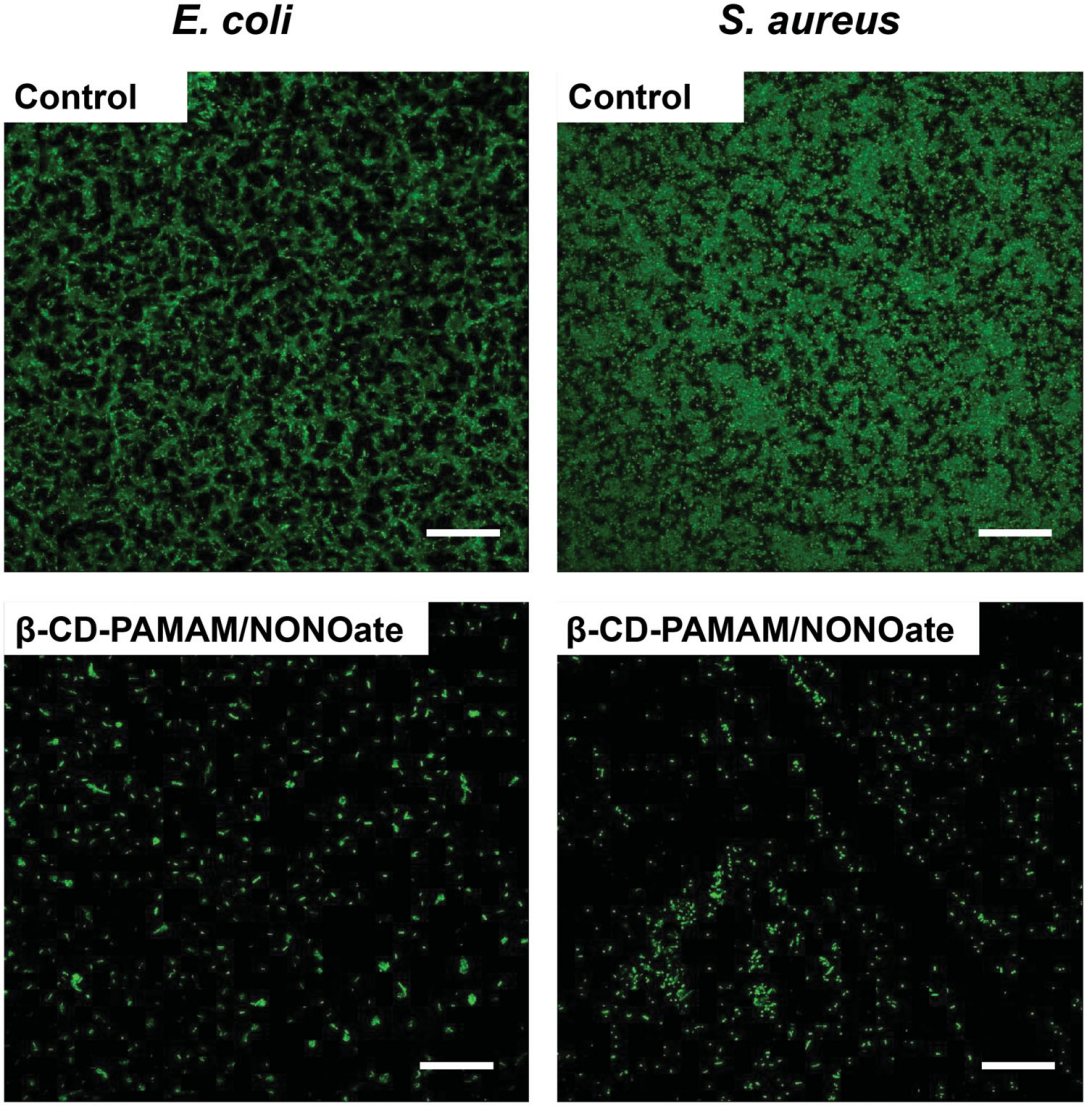
Fluorescence images of *E. coli* (A) and *S. aureus* (B) biofilms stained by SYTO-9 after treated with or without 25 μg/mL β-CD-PAMAM/NONOate for 12 h (scale bar represents 50 μm).

To further confirm the effect of β-CD-PAMAM/NONOate on biofilm, the biofilm dispersal assay was carried out using the *E. coli* and *S. aureus* which had been grown for 24 h and already formed the biofilms. As shown in Figure 4, β-CD-PAMAM showed a little biofilm dispersal effect compared with PBS control, while the NO loading formulations showed the obvious biofilm dispersal effect and a concentration-dependent effect. Quantitatively, β-CD-PAMAM/NONOate at 25 μg/mL resulted in a 65% decrease for *E. coli*, while the 87% decrease at a concentration of 50 μg/mL. The similar results were also confirmed from the data of Figure 5(B) for *S. aureus*. These results indicated that NO played an important role in bacterial biofilm dispersal.

Furthermore, FL and SEM were also employed to evaluate the bacteria biofilm dispersal results intuitively. For FL observation, biofilms were stained by SYTO-9 for fluorescence labeling. As shown in Figure 5, compared with PBS control, the biofilm amount of both *E. coli* and *S. aureus* treated with 25 μg/mL β-CD-PAMAM/NONOate reduced obviously, which confirmed further the excellent dispersal effect on bacterial of the NO system. These results indicated that β-CD-PAMAM/NONOate could disperse biofilm effectively and showed the advantage in anti-biofilm and anti-infection.

### 3.3. Antibacterial activity

**Figure 6.**
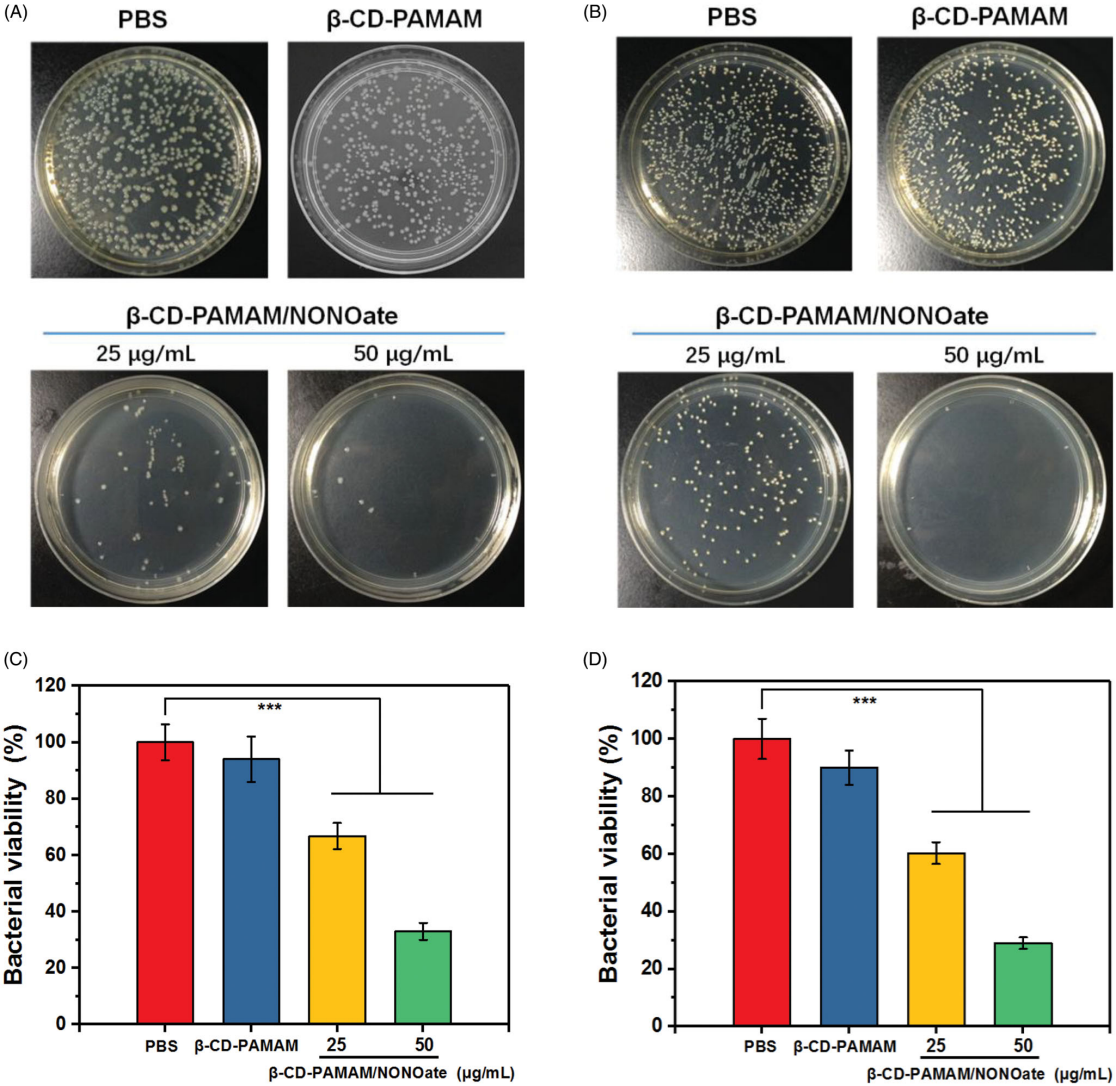
Photographs of bacterial colonies and the quantitative results for *E. coli* (A and C) and *S. aureus* (B and D) treated with different formulations (*n* = 3). The data are presented as the mean ± standard deviations: *** *p* < .001, *n* = 3.

The antibacterial activity of β-CD-PAMAM/NONOate was evaluated by CFU counting assay and the result was shown in Figure 6. The bacteria treated with different formulations were inoculated on culture dishes and were photographed (Figure 6(A,B)). It was found that compared with PBS control and blank β-CD-PAMAM, the formulations of β-CD-PAMAM/NONOate displayed the significantly antibacterial effect, and the antibacterial effect was concentration-dependent. After quantification, it was found that β-CD-PAMAM showed a slight antibacterial effect on both *E. coli* and *S. aureus* at the concentration of 25 μg/mL, and both of them remained more than 90% viability compared with PBS control. The NO loading formulations showed the obvious antibacterial effect on both *E. coli* and *S. aureus*. When treated with 25 μg/mL β-CD-PAMAM/NONOate, the viability of bacterial decreased 33% for *E. coli* and 41% for *S. aureus*, indicating the effectively antibacterial effect. And the formulation of 50 μg/mL β-CD-PAMAM/NONOate induced the more significant antibacterial effect on both *E. coli* and *S. aureus*.

In particularly, *S. aureus* was found to be more sensitive to β-CD-PAMAM and β-CD-PAMAM/NONOate. Cationic polymers can capture negatively charged bacteria and so the antibacterial effect is improved by the positive charge. The cell wall of gram-positive *S. aureus* consisted more negative substances than that of gram-negative *E. coli*, such as peptidoglycans and teichoic acid molecules, and therefore the electronegativity of *S. aureus* is lower than that of *E. coli*. Moreover, the cell wall of *S. aureus* is thicker than that of *E. coli* (Dickson & Koohmaraie, 1989; Beveridge & Graham, 1991). Based on this, we hypothesized that the adsorption of β-CD-PAMAM to *S. aureus* might be stronger than that to *E. coli* and therefore activities of *S. aureus* were more extremely restricted till to death.

**Figure 7.**
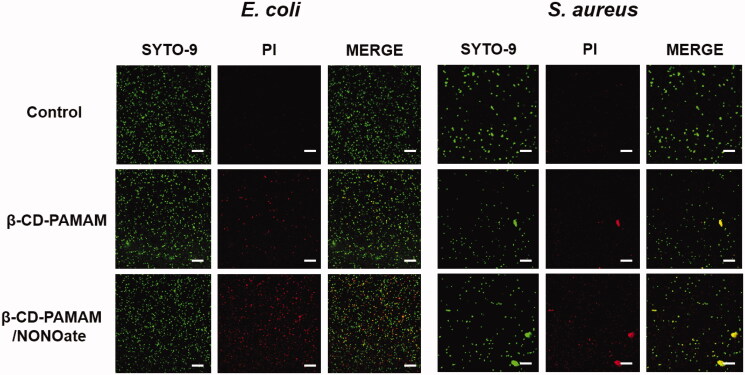
Confocal images of *E. coli* and *S. aureus* stained by SYTO-9 (green) and PI (red) after treated with different formulations (25 μg/mL). SYTO-9 is for live and dead bacteria staining and PI is for dead bacteria staining. Merged image is the merging of the two staining above (scale bar represents 20 μm).

To confirm further the antibacterial effect intuitively, confocal laser scanning microscopy (CLSM) was conducted to indicate the viability by distinguishing living and dead bacteria. Bacteria treated with different formulations were stained by Live/Dead^@^ Baclight^TM^ kit and were observed using confocal laser scanning microscopy. Green fluorescence (SYTO-9) represents the live and dead bacteria and red fluorescence (PI) represents the dead ones. As shown in Figure 7, no red fluorescence was found for the control groups of both *E. coli* and *S. aureus*, indicating bacterial proliferated in a good condition. And the groups treated with β-CD-PAMAM showed a little red spots in PI stain images, implying that β-CD-PAMAM showed some cytotoxicity to bacteria. For the NO loading formulation, obvious red spots could be observed. This result was in accordance with the above results and suggesting the excellent antibacterial effect of NO on both *E. coli* and *S. aureus*.

### 3.4. *In vivo* assays

To confirm the effect of NO-loading materials on CRS therapy, the male SD rats were selected to establish the CRS model using *S. aureus* introduced into their nasal cavities. After that, all animals were divided into 5 groups at random, and treated with different formulations. The Rhinocort, a clinical drug for CRS was set as control.

**Figure 8.**
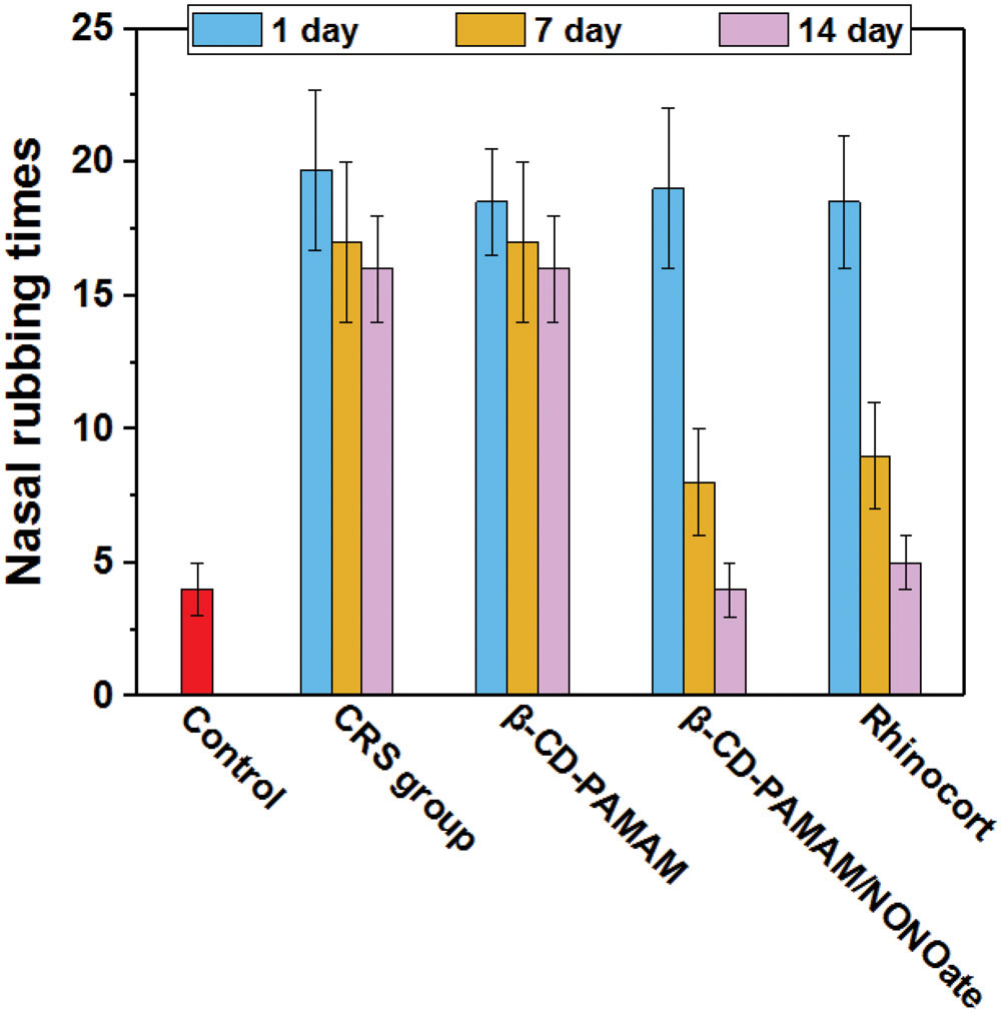
The frequency of nasal rubbing in 10 min in rats treated with different formulations.

It was found that the treatment groups showed significant improvement on the mental status of the rats compared with CRS group and blank β-CD-PAMAM group, implying that the NO was a positive factor on CRS therapy. For the observation of the frequency of nasal rubbing in 10 min for the rats which was shown in Figure 8, the group treated with β-CD-PAMAM/NONOate showed a significant decrease after 7 days treatment, and was no significant difference with that of Rhinocort, indicating the excellent therapy effect on CRS. Moreover, although the blank β-CD-PAMAM showed some antibacterial activity in the above assays, it still showed no significant therapy effect on CRS *in vivo*, which may be resulted from that its weak antibacterial activity and rapid loss *in vivo*.

**Figure 9.**
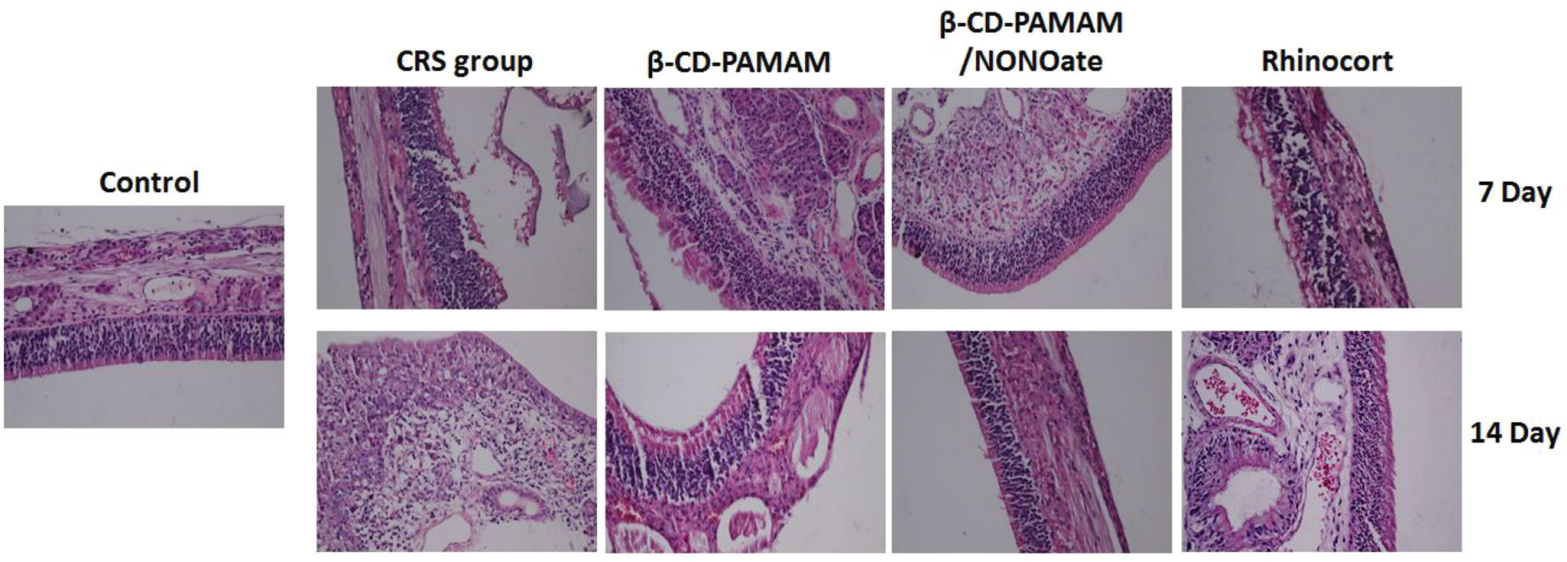
HE staining images of the rat mucosa treated with different formulations.

To further confirm the effect of β-CD-PAMAM/NONOate on CRS therapy, an optical microscope was used to examine the HE staining changes in the mucosa and the result was shown as Figure 9. In the health group (control), there were no obvious inflammatory cell infiltration in epithelium and mucous. However, for CRS group, there were obvious inflammation including many neutrophils, scattered lymphocytes and plasma cells in the mucosa and interstitium. In particular, the mucosa inflammation of the nasal cavity and sinuses somewhat relieved with the time. A large number of neutrophils infiltrated in the mucosa of the nasal cavity and sinuses in the initial stage, with the time, the infiltration of neutrophils in the mucosa mitigated gradually and a large number of lymphocytes and plasma cells infiltrated. The blank β-CD-PAMAM showed little improvement in inflammation after 7-day and 14-day treatment, implying the limited therapy effect on CRS *in vivo*. The β-CD-PAMAM/NONOate and Rhinocort showed the significant therapy effect on CRS showing from the HE images. A few of plasmocytes and lymphocyte could be seen after 7 days treatment of β-CD-PAMAM/NONOate, and no inflammation was observed after 14 days. For Rhinocort treatment, there were a few lymphocytes and plasmocytes in mucosa with a few neutrophils after 7 days, and no inflammation was also observed after 14 days. In summary, the CRS rats showed the local manifestations of nasal inflammation, and the local manifestations of nasal inflammation did not change for the blank β-CD-PAMAM treatment. Compared those, the symptoms did relieve in the rats from nasal steroid group and NO group, especially in those from NO group. These results indicated that the NO-loading materials may play an important role in CRS therapy.

### 3.5. Biocompatibility

**Figure 10.**
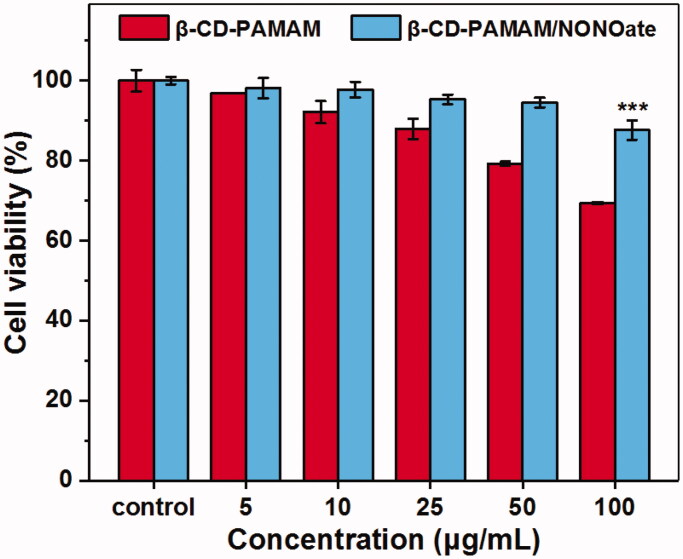
Cell viability of NIH/3T3 treated for 24 h with different formulations at different concentrations (*n* = 5). The data are presented as the mean ± standard deviations: ***p < .001, *n* = 3.

The safety of the biomaterials is the first issue before its application in clinical, then the cytotoxicity of β-CD-PAMAM/NONOate must be studied. The formulations were evaluated on NIH 3T3 cells which provided a suitable model for cytotoxicity evaluation because it played an important role in wound healing, epithelial–mesenchymal interaction and the development of the extracellular matrix (Wong et al., 2007). As shown in Figure 10, it was found that β-CD-PAMAM/NONOate showed the excellent biocompatibility and showed no cytotoxicity at the concentration of 50 μg/mL for 24 h. At a concentration of 100 μg/mL, β-CD-PAMAM/NONOate showed the slight cytotoxicity. In contrary, the blank β-CD-PAMAM showed a concentration-dependent cytotoxicity beyond 25 μg/mL, which may be resulted from the positive charges on the PAMAM. After NO loading, the positive charges decreased and weakened the interaction between materials and the bacterial, and therefore reduced the cytotoxicity.

**Figure 11.**
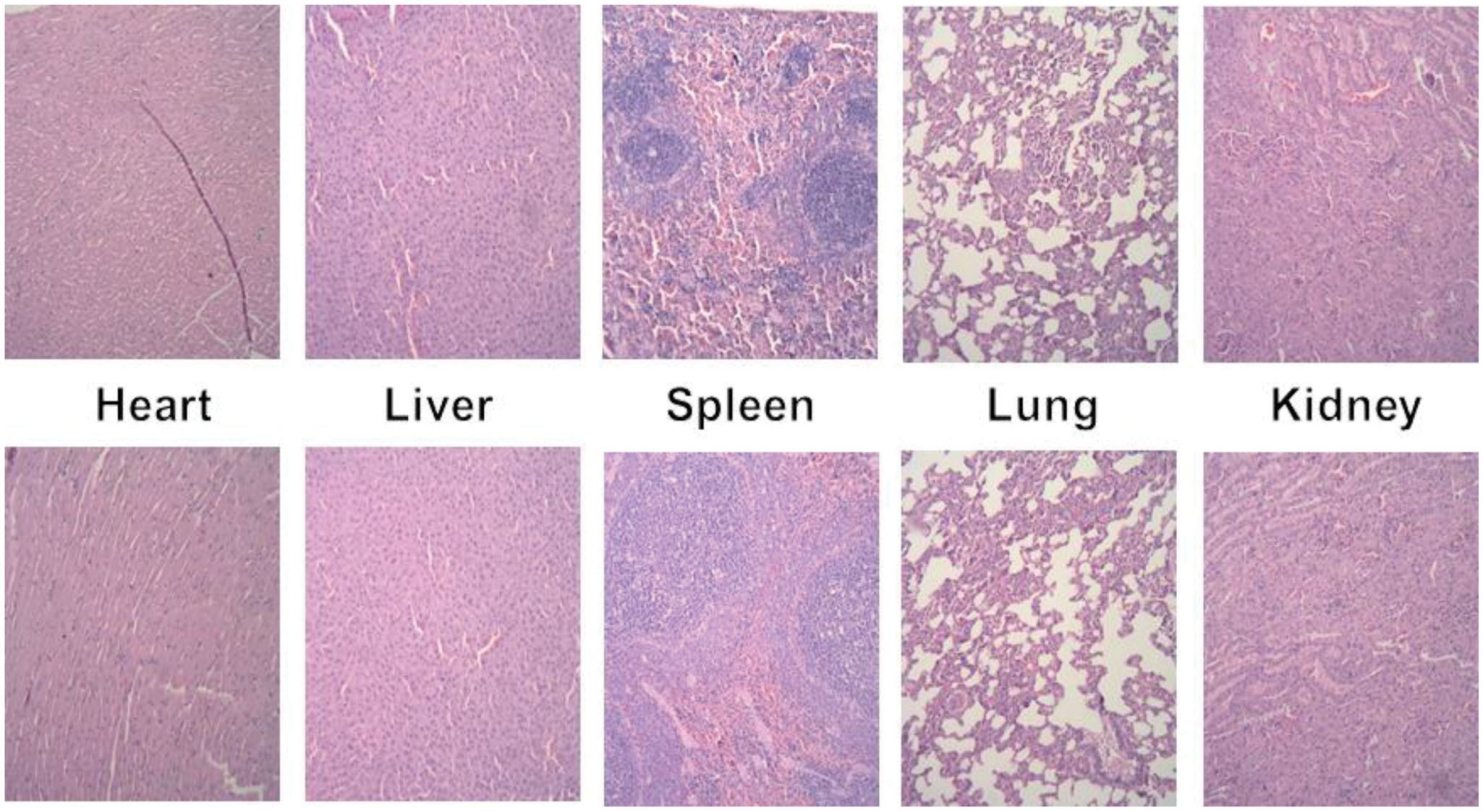
Representative organ histology of rat for control (top row) and formulation (bottom row).

Moreover, the *in vivo* toxicity was also examined, and the result was shown as Figure 11. It was found that no tissue damaged in the viscus (heart, liver, spleen, lung and kidney) of rats in each group according to HE staining results. The result indicated that the NO-loading materials, similar to Rhinocort, mainly acted on the local nasal cavity and has no systematic damage.

## 4. Conclusion

A star copolymer (β-CD-PAMAM) consisting of the β-CD core and seven PAMAM-G3 arms were synthesized to load NO for antibacterial and CRS therapy. NO could be loaded effectively and the obtained β-CD-PAMAM/NONOate showed the good performances on biofilm dispersion and *S. aureus* inhibition, indicating the promising application in CRS treatment. The *in vivo* assay confirmed that β-CD-PAMAM/NONOate displayed excellent therapy effect on CRS and significantly improved the symptoms of the experimental rats, which was no significant different in therapy effect with the clinical Rhinocort. Incorporated with its little toxicity *in vitro* and *in vivo*, the β-CD-PAMAM/NONOate was suggested a promising application in CRS therapy.

## Supplementary Material

Supplemental Material
