# Supplementary material for: Β-Cyclodextrin-graft-poly(amidoamine) dendrons as the nitric oxide deliver system for the chronic rhinosinusitis therapy
Source: Drug Deliv. 2021 Jan 29;28(1):306–18. doi: 10.1080/10717544.2021.1876183 (PMC7850337; doi:10.1080/10717544.2021.1876183)
Supplement: Supplemental Material [file IDRD_A_1876183_SM9937.docx]

***Supporting Information***

β-cyclodextrin-graft-poly(amidoamine) dendrons as the nitric oxide deliver system for the chronic rhinosinusitis therapy

Tao Liu ^a^, Guowei Li ^b^, Xidong Wu ^c^, Shaohua Chen ^a^, Siyi Zhang ^a^, Hong Han ^a^, Hongbin Zhang ^a^, Xiaoning Luo ^a^, Dong Ma *^,b,d^, Xiang Cai *^,e^

^a^ Department of Otolaryngology-Head and Neck Surgery, Guangdong Provincial People’s Hospital, Guangdong Academy of Medical Sciences, Guangzhou, China

^b^ Department of Biomedical Engineering, Jinan University, Guangzhou, China

^c^ Department of Pharmacology, Jiangxi Testing Center of Medical Instruments, Nanchang, China

^d^ MOE Key Laboratory of Tumor Molecular Biology, Jinan University, Guangzhou, China

^e^ Department of Light Chemical Engineering, Guangdong Polytechnic, Foshan, China

* Corresponding authors.

E-mail addresses: [tmadong@jnu.edu.cn](mailto:tmadong@jnu.edu.cn) (Dong Ma), cecaixiang@163.com (Xiang Cai)

**1. Methods of β-CD-PAMAM synthesis**

1.1. Synthesis of per-6-azido-β-CD (β-CD-(N_3_)_7_)

According to the reported method previously by Ashton et al, per-6-azido-β-CD (β-CD-(N_3_)_7_) was synthesized.[1] In brief, Ph_3_P (18.4 g) was dissolved in DMF (100 mL), and then I_2_ (17.8 g) was slowly added over 15min. Dried β-CD (5.7 g) was then added to the resultant dark brown solution when the solution was heated to 50 °C. The mixed solution was stirred under nitrogen atmosphere for 24 h at 70 °C. After that, DMF was removed under the reduced pressure, and the remainder was added into the sodium methoxide (3.0 mol/L in methanol, 30 mL) under cooling with ice-water bath and continued to stir for 30 min. The resultant mixture was dropped into the excess methanol and then dried in a vacuum oven for 2 h. After DMF/methanol recrystallization, β-CD-(I)_7_ was obtained.

For β-CD-(N_3_)_7_ synthesis, NaN_3_ (1.3 g) and β-CD-(I)_7_ (3.8 g) were dissolved in dry DMF (40 mL), and the solution was stirred at 70 °C for 24 h. After that, DMF was removed by the reduced pressure, and the remainder was dropped into excess water. After filtrating, washing with water and drying in a vacuum oven, β-CD-(N_3_)_7_ was obtained as white powder with a yield of 80%. ^1^H NMR (300 MHz, DMSO) for the β-CD-(N_3_)_7_, δ (ppm): 3.15-3.80 (m, 42H, *H*-2, *H*-3, *H*-4, *H*-5, *H*-6), 4.85 (d, 7H, *H*-1), 5.75 (d, 7H, O*H*), 5.90 (d, 7H, O*H*).

1.2. Synthesis of propargyl focal point PAMAM dendron

The propargyl focal point PAMAM (generation = 3) was synthesized according to the method reported by Jae wook lee et al.[2] In brief, propargylamine (1.5 g) was dissolved in methanol (5 mL) under nitrogen atmosphere in an ice-water bath, followed by adding 10 mL of methyl acrylate methanol solution (0.94 g/mL) in a period of 2 h. The mixture was first stirred at 0 °C for 1 h and then stirred at room temperature for 24 h. The unreacted methyl acrylate and methanol were removed by rotary evaporation, and the final product was vacuum dried labeled as PAMAM-D_0.5_ (D_0.5_ means generation 0.5). PAMAM-D_0.5_ (5.36 g) was then dissolved in 20 mL methanol under nitrogen atmosphere in an ice-water bath, followed by adding 30 mL of ethanediamine (21.2 g) methanol solution (0.76 g/mL) in 2 h. The mixture was stirred at 0 °C for 1 h and then stirred at room temperature for 24 h. Subsequently, the unreacted ethanediamine and methanol were removed by rotary evaporation and the final product was vacuum dried labeled as PAMAM-D_1_. Repeated the above two steps twice more until PAMAM-D_3_ was obtained. ^1^H NMR (300 MHz, D_2_O) for the propargyl focal point PAMAM (generation = 3), δ (ppm): 2.20 (t, 1H, -C*H*C-), 2.459 (m, 28H, -C*H*_2_CONH-), 3.29 (m, 28H, -CONHC*H*_2_-), 2.6-3.0 (protons next to amines), 3.42 (d, -CHC-C*H*_2_-).

1.3. Synthesis of the star polymer of β-CD-PAMAM

For β-CD-PAMAM synthesis, β-CD-(N_3_)_7_ (0.52 mmol), PAMAM-D_3_ (4 mmol) and CuSO_4_·5H_2_O (0.8 mmol) were dissolved in distilled water. Subsequently, sodium ascorbate (1.6 mmol) was added under the protection of N_2_. The solution was stirred at 40 °C for 3 d. After the reaction, the mixture was dialyzed for 3 d (MWCO = 2000 Da, USA) and then lyophilized to obtain the star copolymer of β-CD-PAMAM.[3]

**References**

[1] P.R. Ashton, R. Königer, J.F. Stoddart, D. Alker, V.D. Harding, Amino acid derivatives of β-cyclodextrin, The Journal of Organic Chemistry 61(3) (1996) 903-908.

[2] J.W. Lee, J.H. Kim, B.K. Kim, Synthesis of azide-functionalized PAMAM dendrons at the focal point and their application for synthesis of PAMAM-like dendrimers, Tetrahedron Letters 47(16) (2006) 2683-2686.

[3] Q. Lin, Y. Yang, Q. Hu, Z. Guo, T. Liu, J. Xu, J. Wu, T.B. Kirk, D. Ma, W. Xue, Injectable supramolecular hydrogel formed from α-cyclodextrin and PEGylated arginine-functionalized poly (l-lysine) dendron for sustained MMP-9 shRNA plasmid delivery, Acta biomaterialia 49 (2017) 456-471.
